# Functional Anatomical Changes in Ulcerative Colitis Patients Determine Their Gut Microbiota Composition and Consequently the Possible Treatment Outcome

**DOI:** 10.3390/ph13110346

**Published:** 2020-10-28

**Authors:** Anita Bálint, Klaudia Farkas, Orsolya Méhi, Bálint Kintses, Bálint Márk Vásárhelyi, Eszter Ari, Csaba Pál, Tamara Madácsy, József Maléth, Kata Judit Szántó, István Nagy, Mariann Rutka, Péter Bacsur, Diána Szűcs, Zoltán Szepes, Ferenc Nagy, Anna Fábián, Renáta Bor, Ágnes Milassin, Tamás Molnár

**Affiliations:** 1 Department of Medicine, University of Szeged, 6720 Szeged, Hungary; balint.anita@med.u-szeged.hu (A.B.); farkas.klaudia@med.u-szeged.hu (K.F.); tamaramadacsy@gmail.com (T.M.); jozsefmaleth1@gmail.com (J.M.); szanto.kata.judit@med.u-szeged.hu (K.J.S.); rutka.mariann@med.u-szeged.hu (M.R.); bacsurp@gmail.com (P.B.); szdtait@gmail.com (D.S.); szepes.zoltan@med.u-szeged.hu (Z.S.); nagyferenc4703@gmail.com (F.N.); fabiananna9@gmail.com (A.F.); bor.reni86@gmail.com (R.B.); milagn422@hotmail.com (Á.M.); 2 Synthetic and Systems Biology Unit, Institute of Biochemistry, Biological Research Centre, 6726 Szeged, Hungary; orsolyamehi@gmail.com (O.M.); kintses@gmail.com (B.K.); balint.mark.vasarhelyi@gmail.com (B.M.V.); ari.eszter@brc.hu (E.A.); cpal@brc.hu (C.P.); 3 Department of Genetics, Eötvös Loránd University, 1053 Budapest, Hungary; 4 ‘Momentum’ Epithelial Signalization and Secretion Workgroup, Hungarian Academy of Sciences, Department of Medicine, University of Szeged, 1051 Szeged, Hungary; 5 Sequencing Platform, Institute of Biochemistry, Biological Research Centre, 6726 Szeged, Hungary; nagyi@seqomics.hu; 6 HCEMM-BRC Translational Microbiology Lab, 6726 Szeged, Hungary; 7 Department of Biochemistry and Molecular Biology, University of Szeged, 6720 Szeged, Hungary; 8 HCEMM-BRC Metabolic Systems Biology Lab, 6726 Szeged, Hungary

**Keywords:** gut microbiome, faecal microbiome, ulcerative colitis, pouch, familial adenomatous polyposis, functional anatomical changes

## Abstract

Gut microbial composition alters in some special situations, such as in ulcerative colits (UC) after total proctocolectomy and ileal pouch-anal anastomosis (IPAA) surgery. The aim of our study was to determine the composition of the intestinal microbiome in UC patients after IPAA surgery, compared with UC patients, familial adenomatous polyposis (FAP) patients after IPAA surgery and healthy controls. Clinical data of patients, blood and faecal samples were collected. Faecal microbiota structure was determined by sequencing the V4 hypervariable region of the 16S rRNA gene. Overall, 56 patients were enrolled. Compared to the Healthy group, both the Pouch active and UC active groups had higher *Enterobacteriaceae*, *Enterococcaceae* and *Pasteurellaceae* abundance. The Pouch and UC groups showed distinct separation based on their alpha and beta bacterial diversities. The UC group had higher *Prevotellaceae*, *Rikenellaceae*, *Ruminococcaceae* abundance compared to the Pouch active group. Pouch and FAP participants showed similar bacterial community composition. There was no significant difference in the bacterial abundance between the active and inactive subgroups of the Pouch or UC groups. Gut microbiome and anatomical status together construct a functional unit that has influence on diversity, in addition to intestinal inflammation that is a part of the pathomechanism in UC.

## 1. Introduction

The gut microbiome has a great role in some physiological processes in the human body, such as the biosynthesis of vitamins and amino acids, breaking down food compounds, resistance of pathogens, protection against epithelial injury, development and training of immune system, but also to promote angiogenesis, fat storage and modify nervous and immune actions. Not only physiological, but its pathological role is more and more obvious based on human microbiota studies, as well. Most of studies focus on bacterial microbiota composition as a component of the gut microbiota. It contains more than 1000 bacterial species and 100-fold more genes than are found in the human genome [1]. In the healthy gut, most of the bacteria belong to *Firmicutes*, *Bacteroidetes*, *Actinobacteria* and *Verrucomicrobia* [2], and an imbalance of these phyla can drive to dysbiosis. Gut dysbiosis is associated with inflammatory bowel diseases (IBD; ulcerative colitis (UC), Crohn’s disease (CD)) including reduced diversity of bacteria, decreased abundance of *Firmicutes*, *Bacteroides* and increased abundance of *Gammaproteobacteria* [1]. *Enterobacteriaceae* was present in high abundance in clinical and animal-mice-studies [3]. As a member of that family, *Escherichia coli*, especially adherent-invasive *E.coli*, were isolated from biopsies of ileal CD and UC patients [1]. Nevertheless, we can mention other species that were suggested by previous studies as being associated with IBD, such as the adherent and invasive *Fusobacteria*, which mostly colonises the oral cavity and the gut, and it has been shown that it has a higher abundance in active IBD, particularly in colon of UC patients compared to healthy population [1,4]. The The short-chain fatty acids (SCFAs)-producing *Roseburia* is another bacteria that is depleted in IBD [1]. 

The microbiome shows differences in terms of proximal to distal gut^1^. Some special changes have also been reported with regard to the type and localisation of IBD. Microbial composition alters in some special situations like in UC after total proctocolectomy and ileal pouch-anal anastomosis (IPAA) surgery. Inflammation of the pouch, i.e., pouchitis, is the most frequent complication after UC surgery. It occurs in approx. half of the operated patients; moreover, some data suggest that 40% of pouchitis develops within 12 months after surgery [5]. Some studies have suggested that risk for pouchitis after colectomy in UC can be predicted by microbiota composition [6,7]. The connection between dysbiosis and the development of inflammation in UC patients with pouch is not completely understood. The data of inflammation course and dysbiosis after proctocolectomy and IPAA surgery in UC are insufficient to determine the cause and consequence relationship. Some medications, such as anti-inflammatory drug mesalamine, could diminish mucosal inflammation in parallel with decreasing the abundance of *Escherichia* and/or *Shigella* [1]. Data from longitudinal analysis of the IBD microbiome demonstrate that the course of corticosteroids had an influence on microbiome fluctuation compared to bacterial composition of samples from patients who were on a stable dose of medication [8]. However, metronidazole and ciprofloxacin antibiotic course is effective and recommended in majority of pouchitis cases, contrary to UC with an intact colon where data are conflicting and insufficient to verify effectiveness for the maintenance of remission in UC, only if infection is considered [9]. Despite this, familial adenomatous polyposis syndrome (FAP) is a disease with different aetiology, course and outcome, which may require the same surgical procedure as refractory UC. However, pouchitis is uncommon among FAP patients who underwent IPAA formation. Based on the above, we constructed a clinical model of two separate bowel diseases with identical anatomical structure. Our aim was to determine whether alteration in bacterial abundance correlates with the type of disease (UC vs. FAP) and localisation of disease (UC with vs. without pouch). We hypothesised that anatomical variance (i.e., pouch) is associated with the effect that bacterial alterations have on the gut microbiota. Functional anatomical changes may explain differences in the effectiveness of the treatment of intact UC and UC patients with pouch. 

## 2. Results

### 2.1. Demographics and Clinical Data

Overall, 56 participants were enrolled in this prospective, observational, single-centre study: 22 UC patients with a pouch (abbreviated as Pouch (P)), 15 UC patients without a pouch (abbreviated as UC), 6 FAP patients with a pouch (abbreviated as FAP (F)) and 13 healthy volunteers (abbreviated as Healthy (H)). Pouch and UC categories were divided into active (A) and inactive (I) subcategories depending on the inflammatory state of the disease (see definition in the Method section). Samples were obtained from participants who have not been exposed to antibiotics or probiotics for at least 6 weeks prior to study enrolment (see inclusion and exclusion criteria in the Methods and Definitions sections). Demographic and clinical data were comparable between active and inactive Pouch patients. The mean pouch age (time after ileostomy closure) was 5 (SD: 4.6) years. Participants in this study were mostly middle-aged (mean age 45 [SD: 13.9] years). Screening microbiology stool tests excluded the most relevant infectious diseases (e.g., Clostridium difficile, Salmonella) in every enrolled participant. Most of the UC patients without a pouch had left sided or extensive colitis (based on Montreal classification UC distribution was as follows: The E1 type had 2, E2 type had 7 and E3 type had 6 patients). About two-thirds of the Pouch patients had a “mature pouch”, i.e., the time of ileostomy closure was more than 5 years (mature pouch, 63.6%, immature pouch, 36.4%). At the same time, four out of the nine with pouchitis and 10 out of the 13 without pouchitis patients had a mature pouch. Patients were on a stable dose of medication, as detailed in Table 1, with other demographic data. Since antibiotics have a great significance in pouchitis treatment, we analysed the proportion of antibiotic use and its effectiveness, and found that 14 out of 22 Pouch patients (63.6%) had ever received antibiotic therapy due to pouchitis. Eighty-four point five percent of these Pouch patients were antibiotic responders—in other words, they achieved remission after an antibiotic course without need of any immunosuppressant or anti-inflammatory drugs. Regarding serum laboratory parameters, these reflected degree of inflammation (Table 1). The mean levels of inflammatory biomarkers such as serum CRP and faecal calprotectin were higher in active disease, but did not correlate with bacterial composition. Between-group ANOVA on ranks analysis demonstrated significant differences in calprotectin levels (*p* < 0.001), and further testing showed higher calprotectin in Pouch active patients than in Pouch inactive patients (*p* = 0.008) or FAP patients (*p* = 0.008), but not between FAP and Pouch inactive patients (*p* = 0.2).

### 2.2. Bacterial Diversity of the Examined Groups

**Figure 1:**
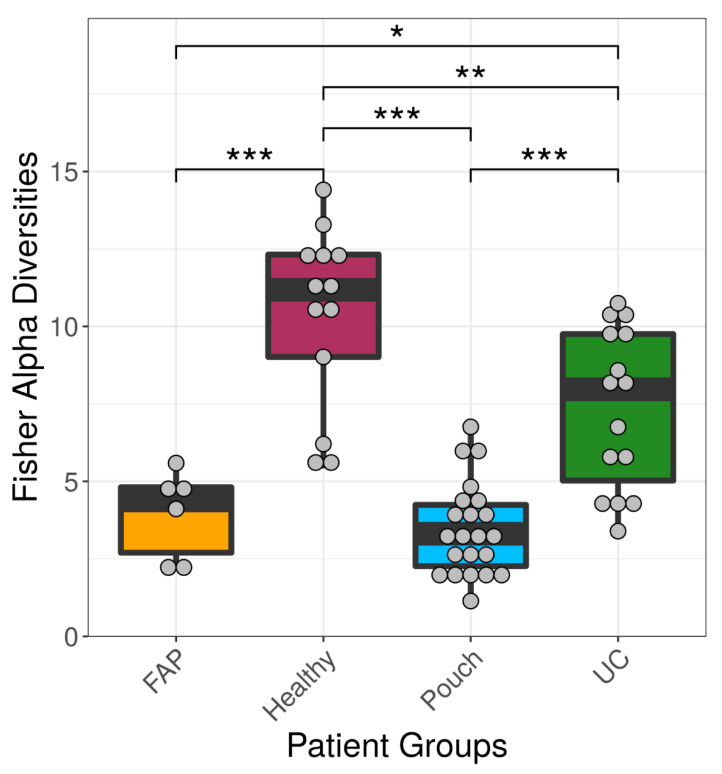
Alpha diversities of the main groups. Data represent Fisher alpha diversity indices at the OTU level based on 16S rRNA profiling of the V4 region. *** indicates significant differences from two-sided Wilcoxon rank sum test *p* = 3.4 × 10^−6^, 0.0008 and 4.4 × 10^−5^ for Healthy versus Pouch, Healthy versus FAP, and Pouch versus UC groups, respectively; ** indicates significant differences from two-sided Wilcoxon rank sum test, *p* = 0.0053 for Healthy versus UC groups; * indicates significant difference from two-sided Wilcoxon rank sum test, *p* = 0.0195 for UC versus FAP groups; sample sizes were 6, 13, 22 and 15 for FAP, Healthy, Pouch and UC groups, respectively. Central horizontal bars represent median values. For details, see Table S2.

**Figure 2:**
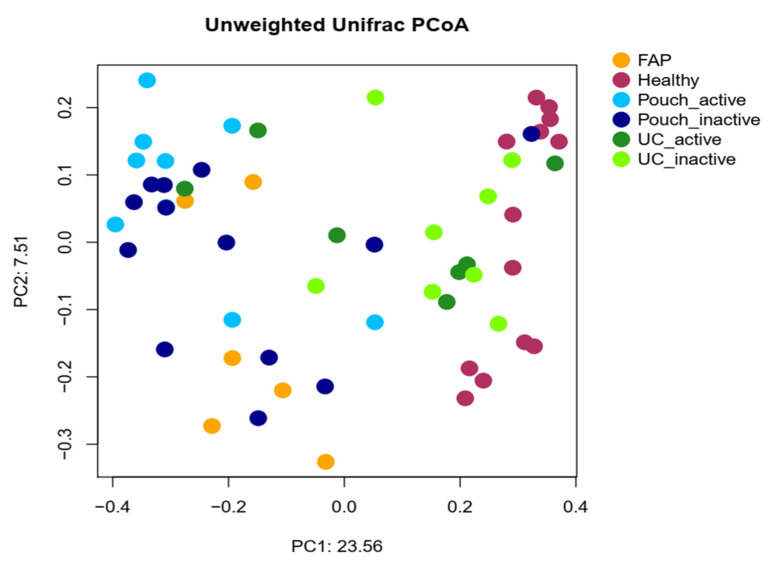
Principal coordinate analysis plot based on unweighted UniFrac distances showing the separation of different groups based on pairwise permANOVA test, except FAP from Pouch active and Pouch inactive groups, Pouch active from Pouch inactive group and UC active from UC inactive group. Sample sizes were 6, 13, 9, 13, 7 and 8 for FAP, Healthy, Pouch active, Pouch inactive, UC active and UC inactive groups, respectively. For details, see Table S3.

**Figure 3:**
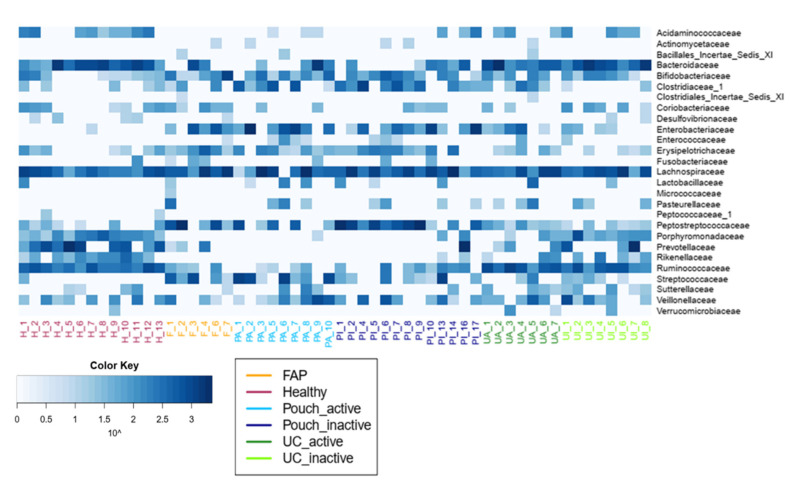
Family level diversity heat map. Bacterial abundances and differences in the abundance of bacterial families between groups are presented in Table S4a and Table S4b, respectively. Sample sizes were 6, 13, 9, 13, 7 and 8 for FAP, Healthy, Pouch active, Pouch inactive, UC active and UC inactive groups, respectively. Abbreviations: H—Healthy, F—FAP, PA—Pouch active, PI—Pouch inactive, UA—UC active, UI—UC inactive.

We examined differences in bacterial profiles between samples of investigated groups with the Wilcoxon rank sum test and found significant separation by diagnosis (Figure 1 and Figure 2). Bacterial families (and OTU categories also) with the greatest changes in alpha diversity differed markedly between Pouch and healthy (*p* < 0.001), UC without pouch and healthy (*p* = 0.002), and FAP and healthy (*p* = 0.0005) (Figure 3). We found decreased alpha diversity in both Pouch and FAP samples compared to UC patients without a pouch (*p* < 0.001 and *p* = 0.02). The healthy group was characterised by the greatest alpha diversity, then decreased variety was observed in UC patients without a pouch, and the lowest diversity was found in Pouch and FAP patients. This separation is presented by beta diversity, as well (Figure 2). Paired permutational multivariate analysis of variance confirm differences between all groups by beta diversity, except between Pouch and FAP groups, which were similar (*p* = 0.09 for FAP vs. Pouch active, *p* = 0.501 for FAP vs. Pouch inactive). 

Microbiome profiles of UC with and without pouch patients were associated with numerous dysbiosis-associated families, including low abundance of the butyrate producing Acidaminococcaceae, Bacteriodeaceae, Porphyromonodaceae, Prevotellaceae, Rikenellaceae, Ruminococcaceae and high abundance of Clostridiaceae, Enterobacteriaceae and Enterococcaceae. Features, with major differences including a decreased abundance of Bacteroidetes phylum, which was largely specific for both UC with and without a pouch. In contrast, the abundance of Gammaproteobacteria was higher in both UC groups than in healthy participants; moreover, it was the highest in both the UC and FAP with a pouch groups. Interestingly, we did not find significant associations of pouchitis with bacterial diversity. Furthermore, focusing on the shift of bacterial families, some taxa were identified by Wilcox test to be less or more abundant in compared groups (Table 2). In samples of Pouch, sequence analysis of bacterial families revealed that Desulfovibrionaceae, Rikenellaceae, Ruminococcaceae were less abundant, and Clostridiaceae, Enterobacteriaceae, Pasteurellaceae, Peptostreptococcaceae and Streptococcaceae were more abundant compared to healthy controls. Although Verrucomicrobiaceae, Rikenellaceae and Ruminococcaceae were more abundant in UC patients without pouch compared to Pouch patients. In both types of IBD samples, Pasteurellaceae showed higher abundance than in healthy samples. Pouch and FAP participants showed similar bacterial community composition, and there was no significant difference in any bacterial family abundance between groups. 

**Figure 4:**
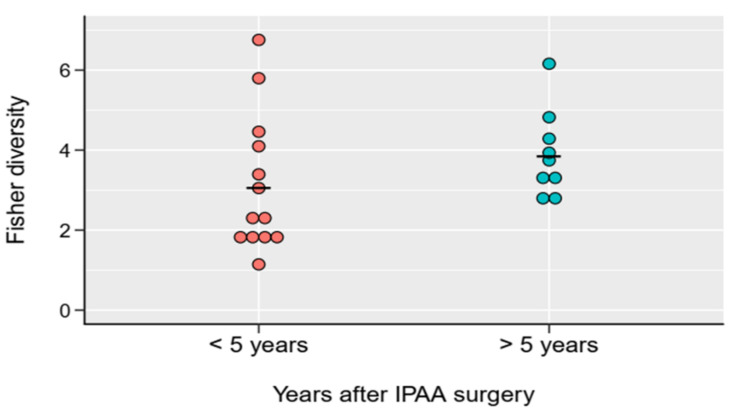
Fisher diversity indices of mature (>5 years elapsed after IPAA surgery) and immature (<5 years elapsed after IPAA surgery) UC pouch (Pouch) samples. No significant difference was observed between the two groups (*p* = 0.2036 from Welch two sample *t*-test. For details, see Table S5_ all_ data final).

Accordingly, during maturation of the pouch, some structural and functional changes develop, and thus there may be some alterations in local microbiota as well. Even so, we did not find any major differences in beta diversity between mature and immature pouches (Figure 4). 

## 3. Discussion

We found that bacterial diversity of healthy controls and subtypes of UC formed distinct bunches; moreover, the non-IBD colectomised group was closer to colectomised UC patients than healthy controls by principal coordinates analysis of unweighted Unifrac distances. As suggested in previous studies, large differences were found in alpha and beta diversity between healthy and UC patients. Shifts in participants with UC mirrored earlier observations of dysbiosis, relative reduction in *Acidaminococcaceae*, *Bacteriodeaceae*, *Porphyromonodaceae*, *Prevotellaceae*, *Rikenellaceae*, *Ruminococcaceae*, and higher abundance of *Clostridiaceae*, *Enterobacteriaceae* and *Enterococcaceae*. These observations are consistent with the results of a study [10] which recruited 140 pouch patients that show that bacterial diversity in UC patients is lower than in healthy subjects. Reshef et al. in this study [10] noticed little difference in microbiota composition between UC with non-pouchitis, UC with pouchitis and FAP with pouch samples. They did not find diversity difference between samples of UC with non-pouchitis, FAP with pouch and UC without pouch. However, it should be noted that in further analysis, they discovered a reduction in *Faecalibacterium* in patients with UC pouchitis compared to UC non-pouchitis and FAP pouch participants [10]. Li et al. found less diverse microbiota in patients with UC and UC with pouch compared to healthy samples, and likewise in our study, patients with a pouch had an altered microbiota composition compared with UC patients [11]. 

Halfvarson et al. calculated the mean distance of taxa from each observed IBD sample to the healthy plane and revealed that all subtypes of IBD significantly deviated from healthy samples [8]. The colonic CD and UC samples were closer to the healthy plane than ileal CD samples and the highest volatility was observed for ileal CD patients who had underwent ileocoecal resection. The authors explained that the high volatility was due to the removal of the ileocoecal valve and altered intestinal physiology. Their results suggest the importance of disease localisation/extension, whether is it colonic or ileal or resected gut. In our study, Pouch and FAP samples showed high similarity linked to bacterial diversities. On the other hand, both Pouch and FAP samples significantly differed from healthy and UC without pouch samples, as well. Shifts in colectomised participants, both with UC or FAP, showed similar differences, namely, depletion of *Bacteroidetes* and enrichment of *Firmicutes* and *Proteobacteria*. We think that differences were largely driven by altered anatomical conditions as a result of surgery. Moreover, based on our results, it seems that anatomical status may be as significant as disease type itself or the presence of pouchitis. However, some data from previous prospective studies demonstrate that UC patients with a pouch had diminished diversity compared to FAP patients with pouch [5,12]. We identify some bacterial families specific for UC and colectomised participants that verify dysbiotic environmental of gut. Microbiome varies along the human gastrointestinal (GI) tract, which justifies the study by Vasapolli et al. where the predominant genera in the upper GI tract (*Gemella, Veillonella, Neisseria, Fusobacterium, Streptococcus, Prevotella, Pseudomonas and Actinomyces*) were almost absent from the lower GI tract, where the microbial communities mainly comprised *Faecalibacterium*, *Ruminococcus*, and *Bacteroides* [13]. Another example for the importance of localisation is that more and more data are available about intestinal microbiome divergence between ileal and colon CD [4,8,14]. Naftali et al. suggested different underlying mechanism due to the highly significant separation of the microbiome of ileal and colon CD samples which was unaffected by the biopsy’s location, inflammatory state or patient’s condition (i.e., remission or relapse) [4]. They observed higher microbial dysbiosis index values in ileal samples compared to colon, meaning more severe dysbiosis in those patients. A study group from Israel published their gene expression-investigation findings about no alteration in gene expression in UC patients with normal ileal mucosa; however, IPAA surgical intervention was associated with some molecular changes. Interestingly, these changes were observed in patients with FAP who underwent IPAA surgery. It should be added that more significant gene expression changes were among UC patients with a pouch than in FAP patients with a pouch. In this study, UC, non-pouchitis UC, pouchitis UC and Crohn’s disease-like pouchitis showed a spectrum of molecular changes associated with disease phenotypes and the severity of inflammation [15]. 

Studies suggest that some bacterial species predict pouchitis [16] even before total colectomy and IPAA formation and thus this may be an opportunity to express an outcome [5]. A study from Leuven indicated that the predominant presence of *Ruminococcus gnavus*, *Bacteroides vulgatus* and *Clostridium perfringens* and absence of *Blautia* spp and *Roseburia* spp in faecal samples of patients with UC before colectomy increased the risk of pouchitis [6]. In another work, the authors found an association between pouchitis and decreased *Ruminococcus*, *Lachnospira* and *Coprococcus* genera [17]. A number of studies showed the difference by diversity between pouchitis and non-pouchitis patients supporting the association between inflammation and dysbiosis. As an element of dysbiosis, reduction in *Ruminococcaceae* was noticed in our investigation, as well. *Lactobacillus*, *Bifidobacterium* and *Faecalibacterium* have been shown to be protective by the stimulation of anti-inflammatory cytokine production and down-regulation of inflammatory cytokines [1]. *Faecalibacterium prauznitzii* was underrepresented in specimens of IBD patients; moreover, some studies reported lower abundance in ileal CD than in colon CD samples [4] and higher rates of relapse of post-surgery CD [1]; restitution of *F.prauznitzii* and maintenance of remission in UC showed connection [1]. The reason for this could be that the *Faecalibacterium* genus belongs to *Ruminococcaceae*, which has a role in butyrate production. The short-chain fatty acids’ (SCFAs) beneficial effects (such as being the primary energy source of colonic epithelial cells) on the intestinal mucosa have been verified [1]. Some studies confirmed no significant differences between the bacterial cultures of pouchitis and non-pouchitis patients [18,19]. However, not all papers have been able to verify these findings [20], demonstrating the complexity of the topic. 

In general, *Firmicutes* and *Bacteroidetes* play an important role in influencing dysbiosis that could be associated with the modulation of immune response; however, evidence is conflicting. We verified the lower abundance of *Bacteroidetes* and higher abundance of *Firmicutes* and *Proteobacteria* in Pouch patients when compared to heathy participants. In addition, *Bacteroidetes* were more decreased related to active UC pouch patients than in inactive UC patients, which suggests an association between low abundance of *Bacteroidetes* and inflammation; in the same way, *Bacteroidetes* had lower abundance also in FAP patients compared to inactive UC patients, which represents a connection with gut structural modification, i.e., decreased host carrying capacity besides IBD or abnormal immune response-related microbial alterations. *Clostridium* and *Bacteroides* species could facilitate the expansion of T reg cells that attenuate intestinal inflammation [1]. *F. prauznitzii*, *Clostridia* and *B.fragilis* could mitigate the severity of colitis in some mice models [1]. Besides that, data from other studies analogous with our results confirmed only limited differences between pouchitis and non-pouchitis in a matter of bacterial composition [21]. A previous study suggested a relative increase in *Proteobacteria*—more precisely, *Proteobacteria* are able to sustain a constant density in the individuals with IBD, while the remaining phyla decrease in density [22]. Corresponding to the literature, the abundance of *Proteobacteria* was higher in both active and inactive UC patients compared to healthy controls in our study. Nevertheless, *Proteobacteria* is the typical phylum that exists in the small intestine where transit is faster than in the colon, and simple sugar and amino acid metabolism is favoured, thus rapidly dividing facultative anaerobe communities dominate; whereas the slow flow colon, where metabolism is dominated by the fermentation of complex polysaccharides, results in a great diversity of species (e.g., *Bacteroidales, Clostridiales*) [23]. In accordance with that knowledge, in our study, *Proteobacteria* was more abundant in both Pouch and FAP patients, and we found more *Bacteroidales* in UC samples than in samples of colectomised patients. 

If associations exist between low bacterial diversity and inflammation, thus it would be logical to be a relationship between low bacterial diversity and elevated inflammatory markers. Similarly to our result, Halfvarson et al. [8] observed higher faecal calprotectin concentrations in IBD patients compared to healthy controls; however, they did not found correlation between faecal calprotectin and distance from the healthy plane, as we did not detect any connection between calprotectin and bacterial diversities. 

We need to take into account that the mechanism of gut dysbiosis related to IBD is not only driven by inflammation-associated microbiota alterations, because this view is challenged by data which proved that dysbiosis exists in patients with remission. Microbiota density is influenced by both the host’s carrying capacity and the fitness of the microbiota. Carrying capacity—that is, the maximal density of organisms supported by an ecosystem—depends on environmental resources and the ability to effectively utilise the available sources [22]. Significantly decreased microbiota diversity suggests that an altered gut structure (total proctocolectomy and IPAA formation) has a large influence on carrying capacity. Some data suggest that the microbiome composition of the pouch changes over the time (shifts from “ileal to colonic type”) [7,24,25]. Nonetheless, diversity differences between colectomised and non-colectomised patients suggest that pouches do not offer the same colonisation conditions (even after years of surgery) as that the large intestine environment provides. Although we did not find statistically significant differences between mature and immature UC pouch gut microbiomes, our data suggest the dominance of *Bacteroideaceae* in mature pouches, which is in line with the literature [10,24,25]. 

As mentioned above, responses to antibiotic therapy have a great significance in UC pouchitis treatment. The logical question is whether patients who did not respond to antibiotic therapy have an altered bacterial composition compared to antibiotic responders. The combination of antibiotics, namely metronidazole and ciprofloxacin, can improve the outcome of the majority of pouchitis cases, and only in 10–15% of cases does chronic, treatment refractory pouchitis develop [9]. This proportion was comparable to our results. However, the long-term use of antibiotics may induce antibiotic resistance [2]. An antibiotic course is associated with an overall reduction in bacterial richness [5,22]. In the study by Reshef et al. [10], samples from patients treated with chronic antibiotic therapy showed lower diversity than samples from participants treated with immunomodulators or biologic therapies. The decreased bacterial diversity that was seen in our Pouch cohort cannot only be explained by high rates of antibiotic use among colectomised UC patients. The manipulation of gut microbiota with probiotics and prebiotics is an obvious and practical strategy in the management of UC with minimal adverse events. Some studies proved the induction of remission and prevention of relapse with probiotics in the case of pouchitis, especially VSL#3, with mild to moderate disease activity. On the other hand, the role of prebiotics is unclear [26,27]. 

Our study has some strengths and limitations. The first limitation is the relatively small number of enrolled participants, although it corresponds with the literature in this topic. The second limitation is that 16S RNA gene sequencing technique represents gene copy number, not true bacterial counts; however, this methodology is currently the most widespread and used in investigations of gut bacterial composition. The third limitation is that we did not examine the endoscopic and histological findings of patients. Endoscopies were carried out only on a limited number of patients. Disease activity was defined by clinical and laboratory parameters in order to avoid colonoscopy as an invasive intervention. A strength of our study was the multi-group analysis: we compared data of multiform types of UC, FAP and healthy participants, which allows for testing multiple relationships. 

## 4. Materials and Methods 

### 4.1. Patients

Active and inactive UC patients who underwent restorative proctocolectomy and IPAA formation (“Pouch active and Pouch inactive”) and were treated at Department of Medicine, University of Szeged were enrolled in the study. The enrollment period was between 31 January 2017 and 30 November 2018. The sampling technique was consecutive sampling, therefore we enrolled every patient who met the inclusion criteria during the enrollment period. For comparison, active and inactive UC patients with different locations/extents treated at 1st Department of Medicine, University of Szeged, were also enrolled (“UC active and UC inactive”). The other group of patients that was compared with the main group (that is, UC patients with a pouch) was patients with FAP who underwent proctocolectomy and IPAA formation (“FAP”). The same number of healthy subjects were enrolled as a control group. All of the patients and healthy volunteers who agreed to participate in the study signed an informed consent form. 

Clinical data of patients, blood and faecal samples were collected to determine C-reactive protein (CRP), leukocyte and thrombocyte count, serum iron, haematocrit, haemoglobin, faecal calprotectin concentration, and microbiota composition. Faecal samples were stored at −20 °C until processing. Subjects’ serums were analysed after sampling. Patients were assessed depending on disease phenotype, clinical activities and type of concomitant therapy. The response to drug treatment was also evaluated, especially the response to antibiotic therapy in the case of UC patients with a pouch. Disease activity was assessed by the clinical subscore of the Pouchitis Disease Activity Index (PDAI) [28] and clinical subscore of the Mayo score (partial Mayo Score) [29]. 

### 4.2. Definitions 

Inclusion criteria: Definitive UC for at least 3 months duration before enrolment, concomitant treatments continued during the study period, such as 5-aminosalicylate and/or a corticosteroid, and/or an immunosuppressant was permitted at a stable dose for at least 8 weeks prior to inclusion.

Exclusion criteria: age below 18 years, antibiotic or probiotic or gastric acid inhibitor use 6 weeks before inclusion, steroid titration up to 6 weeks prior to study entry, chronic NSAID (nonsteroidal anti-inflammatory drugs) use or NSAIDS within 6 weeks of study recruitment (apart from 5-ASA therapy), pregnancy, any other inflammation of bowel than UC (for example diverticulitis or infectious colitis), colon tumour, any acute or chronic severe disease especially bowel and autoimmune diseases, active infection, ano-rectal cuff stricture, insufficiency of IPAA, fistulas, inability to give informed consent and if the patient withdrew consent.

We defined the inflammation of the pouch (active) as ≥2 points in clinical subscore of the PDAI. Remission (inactive) was defined as 0–1 subscore. In a case of UC, active disease was determined as ≥2 points of partial Mayo Score. Inactive disease was determined as 0–1 points of partial Mayo score.

Definition of “mature pouch”: Time after ileostomy closure was more than 5 years. Immature pouch: time after ileostomy closure was less than 5 years. 

### 4.3. Specimen Collection and Storage

Serum and faecal specimens for research were collected during enrolment time point. Serum was analysed for CRP, complete blood count, serum albumin and iron level right after sampling. Following defecation, stool samples were immediately placed into commercial 8 mL plastic tubes (Biolab^®^, Budapest, Hungary) without buffer and tubes were transferred as soon as possible. If necessary, the sample could be stored in the refrigerator until transport (±4 °C). Faecal samples were frozen within 4 h and stored at −20 °C until processing for the determination of calprotectin levels and microbiome profiles. All frozen samples were processed within 6 months. One more stool specimen was sent for microbiological tests to exclude infectious agents. 

### 4.4. 16S rRNA-Based Bacterial Community Profiling

To determine the faecal microbiota composition for each individual involved in the study, we sequenced and analysed the V4 hypervariable region of the 16S rRNA genes from faecal samples. The metagenomic DNA was extracted from faecal samples by using ZR Fecal DNA MiniPrep™ kit (Zymo Research, Irvine, CA, USA) following the manufacturer’s instructions. For DNA isolation, we needed ≤ 150 mg faecal sample/individual. After donation, faecal samples were stored at −20 °C until DNA isolation was performed. DNA concentration was determined for each sample using a fluorometric method (Qubit dsDNA BR Assay Kit, Thermo Fischer Scientific, Waltham, MA, USA). Extracted DNA was stored at −80 °C.

The V4 region of the 16S rRNA gene was PCR amplified with dual-indexed Illumina primer pairs, using different combinations of barcoded forward and reverse primers (v4.SA501-508 and v4.SA701-707, respectively, Table S1) as previously described [30,31]. The primers consist of the appropriate Illumina adapters, an 8 nt index sequence, a 10 nt pad sequence, a 2 nt linker and specific sites for the V4 region. The PCR reactions consisted of 1.5 μL (30 ng) of template DNA, 10 μL of Phusion HF buffer (Thermo Fisher Scientific), 4 μL of 2.5 mM deoxynucleotide triphosphates mix (dNTPs), 0.5 μL of Phusion DNA polymerase (2 U µL^−1^) (Thermo Fisher Scientific), 1–1 μL of primers, 10 μM each, 3 μL DMSO (100%) and 29 μL of nuclease-free H_2_O in a final reaction volume of 50 μL. The following thermocycler conditions were used: 95 °C for 2 min, 25 cycles of 95 °C for 20 s + 56 °C for 15 s + 72 °C for 30 s and 72 °C for 10 min. Following gel electrophoreses of the PCR products, the 400 bp amplicons were extracted from the gel (Thermo Fischer Scientific GeneJET Gel Extraction Kit) and, following a second purification step (Zymo Research DNA Clean and Concentrator-5 Kit), were sequenced using the MiSeq Illumina platform. To prepare the samples for sequencing, the amplicons were quantified using a fluorometric method (Qubit dsDNA BR Assay Kit, Thermo Fischer Scientific) and libraries were mixed with Illumina PhiX in a ratio of 0.95:0.05. Sequencing on the Illumina MiSeq instrument was carried out with a v2 500 cycle sequencing kit (Illumina). Then, 100 µM stock custom sequencing primers were mixed with standard read1, index read and read2 sequencing primers included in the MiSeq cartridge.

After sequencing, 16S rRNA reads were demultiplexed and processed with the Mothur software (version 1.42.0) There were 20,204 average counts per sample. To filter out the low read counts, we followed the protocol of Rettedal, 2014 [32]. The number of sequences per sample was equalised to 3000 read counts using random re-sampling with a custom R script. Sequences were merged at the level of 97% sequence identity and taxonomically assigned using the Silva ribosomal RNA database [33]. After removal of reads that could not be classified, 1689 OTUs remained.

To quantify within-group (e.g., Healthy group, FAP group) diversity from 16S rRNA data, we used the *vegan* R package to calculate the most commonly used alpha diversity indices [34,35] (Fisher index, Figure 1 and Figure S1; Shannon and inverse Simpson indices, Figure S2, Table S2). Unweighted Unifrac distances (Figure 2 and Table S3) were computed with Phyloseq (version 1.22.3 R package) [36] and pairvise permANOVA analysis was calculated with the *pairwiseAdonis* R package.

To identify differentially abundant taxa between different groups OTUs (operational taxonomic unit) were classified at the family level because the V4 region allows accurate identification only down to this level [37] (Table S4a). We applied *edgeR* (version 3.16.5 R package) [38] as suggested previously [39]. To this end, abundances were normalised using the TMM (trimmed mean of M-values) method [40] and then different groups were compared using negative binomial tests in a pairwise manner. We used the Benjamini–Hochberg false discover rate correction method to correct the *p* values for multiple testing [41] (Table S4b).

### 4.5. Ethics

Ethical approval was received from the Medical Research Council, Scientific and Research Committee and the Regional and Institutional Human Medical Biological Research Ethics Committee, University of Szeged (approval No.: 49/3-64/007; 42/2017-SZTE). All patients and volunteers were informed of the study procedure. Their written informed consent was obtained.

### 4.6. Data analysis

To determine statistical differences between groups the following tests were used: Welch two sample *t*-test, Wilcoxon rank sum test, pairwise permANOVA test and pairwise two-sided negative binomial test. Statistical differences between groups for faecal calprotectin levels were determined by the Mann–Whitney rank sum and Kruskal–Wallis one-way ANOVA on ranks test (normality test failed). Descriptive parameters are shown as mean with standard deviation (SD); except for faecal calprotectin was shown as median with minimum-maximum values. *p*-value < 0.05 was considered as significant. 

## 5. Conclusions

The results of this study demonstrate a marked shift in gut microbiota in UC patients with pouch. We verified significant difference in diversity between IBD and non-IBD groups, although colectomised UC and FAP patients showed similar bacterial composition. In this line, gut microbiome and its localisation together construct a functional unit that could be an explanation of our results. More animal, transitional and clinical studies are needed with sophisticated molecular microbiologic techniques to understand the complex networking and function of gut microbiome and its role in UC with IPAA formation. 

## Figures and Tables

**Table 1:** Patients’ demographic, clinical and biochemical features.

	Ulcerative colitis (UC)			UC-Pouch (P)			FAP (F)	Healthy (H)
	Active	Inactive	Total	Active	Inactive	Total		
No. of patients	7	8	15	9	13	22	6	13
Gender (male/female), No. of patients	6/1	5/3	11/4	4/5	8/5	12/10	3/3	6/7
age at inclusion (SD, years)	48.6 (21.7)	45.1 (14.6)	46.7 (17.6)	51.5 (13.9)	41.3 (12.8)	45(13.9)	31.7 (6.5)	32.35 (7.45)
disease duration at inclusion (SD, years)	10.9 (11.1)	11.4 (9)	11.1 (9.6)	14 (6.7)	15.1 (10.5)	15 (9)	11.9 (7.3)	NA
time after IPAA surgery (SD, years)	NA	NA	NA	4.6 (4.2)	5 (5)	5 (4.6)	8.7 (7.4)	NA
Therapy (No. of patients)								
none	1	0	1	2	9	11	6	13
oral 5-ASA	3	6	9	0	0	0	0	0
topical 5-ASA	1	3	4	2	0	2	0	0
oral corticosteroid	3	2	5	1	0	1	0	0
topical corticosteroid	2	2	4	0	0	0	0	0
azathioprine	2	4	6	0	0	0	0	0
biological therapy (IFX, ADA)	1	0	1	0	0	0	0	0
Laboratory parameters								
faecalcalprotectin (median, min–max, μg/g)	1000 (116.4–1000)	303.4 (12.6–1000)	360.2 (12.6–1000)	1000 (38.8–1800)	428 (100–1222)	691.7 (38.8–1800)	284.8 (145.1–711.7)	11.8 (6.9–20.5)
CRP (mean, SD, mg/L)	17.8 (17.5)	7.7 (5.1)	12.7 (13.5)	9.3 (5.1)	7.3 (4.6)	7.8 (4.6)	2.1 (0.2)	NA
serum iron (mean, SD, μmol/L)	10.9 (7.3)	16.6 (5.7)	13.7 (7)	9.1 (8.5)	12 (6.8)	11 (7.3)	NA	NA
haematocrit (mean, SD, L/L)	39 (5.5)	42.3 (5)	40.6 (5.3)	38 (4.4)	42.9 (3.5)	41(4.5)	42 (3.3)	NA
thrombocytes (mean, SD, G/L)	335.4 (93.4)	262 (51.4)	298.7 (81.9)	349 (121.1)	291.3 (73.5)	315.6 (97.8)	309.5 (162.7)	NA
albumin (mean, SD, g/L)	42.4 (5.3)	47.3 (3.7)	44.9 (5.1)	42 (6.9)	46.8 (2.9)	45.3 (4.8)	41.5 (2.1)	NA

NA—not applicable; IPAA—ileal pouch-anal anastomosis; 5-ASA-5-aminosalycilic acid.

**Table 2:** Differences in bacterial family abundance between compared groups. Some taxa were identify to less or more abundant in compared groups. Only significant shifts are presented.

Compared Groups	Higher or Lower Abundance Group	Taxonomic Annotation	Adjusted *p* Value
Healthy vs. Pouch active	less abundant in Pouch active	*Acidaminococcaceae*	0.032
		*Porphyromonadaceae*	0.007
		*Prevotellaceae*	<0.001
		*Rikenellaceae*	<0.001
		*Ruminococcaceae*	0.007
	more abundant in Pouch active	*Actinomycetaceae*	0.040
		*Clostridiaceae*	0.016
		*Enterobacteriaceae*	<0.001
		*Enterococcaceae*	0.011
		*Pasteurellaceae*	0.007
		*Streptococcaceae*	<0.001
Healthy vs. Pouch inactive	less abundant in Pouch inactive	*Bacteroidaceae*	0.018
		*Desulfovibrionaceae*	0.018
		*Porphyromonadaceae*	0.018
		*Rikenellaceae*	0.002
		*Ruminococcaceae*	0.037
	more abundant in Pouch inactive	*Clostridiaceae*	0.018
		*Enterobacteriaceae*	<0.001
		*Pasteurellaceae*	0.018
		*Peptostreptococcaceae*	0.004
		*Streptococcaceae*	0.018
Pouch active vs. UC inactive	less abundant in UC inactive	*Clostridiaceae_1*	0.04
	more abundant in UC inactive	*Acidaminococcaceae*	0.01
		*Porphyromonadaceae*	<0.001
		*Prevotellaceae*	<0.001
		*Rikenellaceae*	<0.001
		*Ruminococcaceae*	<0.001
Pouch active vs. UC active	more abundant in UC active	*Prevotellaceae*	0.015
		*Rikenellaceae*	0.020
		*Ruminococcaceae*	0.015
		*Verrucomicrobiaceae*	0.015
Pouch inactive vs. UC active	more abundant in UC active	*Verrucomicrobiaceae*	0.031
Pouch inactive vs. UC inactive	less abundant in UC inactive	*Peptostreptococcaceae*	0.029
Healthy vs. UC active	more abundant in UC active	*Pasteurellaceae*	0.011
		*Enterobacteriaceae*	<0.001
		*Enterococcaceae*	0.041
Healthy vs. UC inactive	more abundant in UC inactive	*Pasteurellaceae*	0.042
		*Bacillales_Incertae_Sedis_XI*	0.039
		*Enterobacteriaceae*	<0.001
		*Micrococcaceae*	0.049
		*Pasteurellaceae*	<0.001
		*Peptostreptococcaceae*	0.001
		*Streptococcaceae*	0.001
	more abundant in healthy	*Acidaminococcaceae*	0.004
		*Porphyromonadaceae*	0.002
FAP vs. UC inactive	less abundant in UC inactive	*Peptostreptococcaceae*	0.019
	more abundant in UC inactive	*Porphyromonadaceae*	0.009
		*Rikenellaceae*	0.040

## Supplementary Materials

The following are available online at https://www.mdpi.com/1424-8247/13/11/346/s1, Figure S1: Alpha diversity differences between all groups. Data represent Fisher alpha diversity indices at the OTU level based on 16S rRNA profiling of the V4 region. No significant difference was observed between Pouch_active and Pouch_inactive, and UC_active and UC_inactive groups. Sample sizes were 6, 13, 9, 13, 7 and 8 for FAP, Healthy, Pouch active, Pouch inactive, UC active and UC inactive groups, respectively. Central horizontal bars represent median values. For details, see Table S2. Figure S2: Shannon (a) and inverse Simpson (b) alpha diversity indices at the OTU level based on 16S rRNA profiling of the V4 region. Sample sizes were 6, 13, 9, 13, 7 and 8 for FAP, Healthy, Pouch active, Pouch inactive, UC active and UC inactive groups, respectively. Central horizontal bars represent median values. For details, see Table S2. Table S1: Primer sequences, Table S2: Diversity values and Wilcoxon test results. Table S3: Pairwise permANOVA results. Table S4a: Bacterial abundance; Table S4b: Differential abundance. Table S5: Patients all data final.
