# Supplementary material for: Functional Anatomical Changes in Ulcerative Colitis Patients Determine Their Gut Microbiota Composition and Consequently the Possible Treatment Outcome
Source: Pharmaceuticals (Basel). 2020 Oct 28;13(11):346. doi: 10.3390/ph13110346 (PMC7692875; doi:10.3390/ph13110346)
Supplement: Supplementary file 1 [file pharmaceuticals-13-00346-s001.zip › Faecal microbiota composition of pouch_Figures_V3.pptx]

## Slide 1
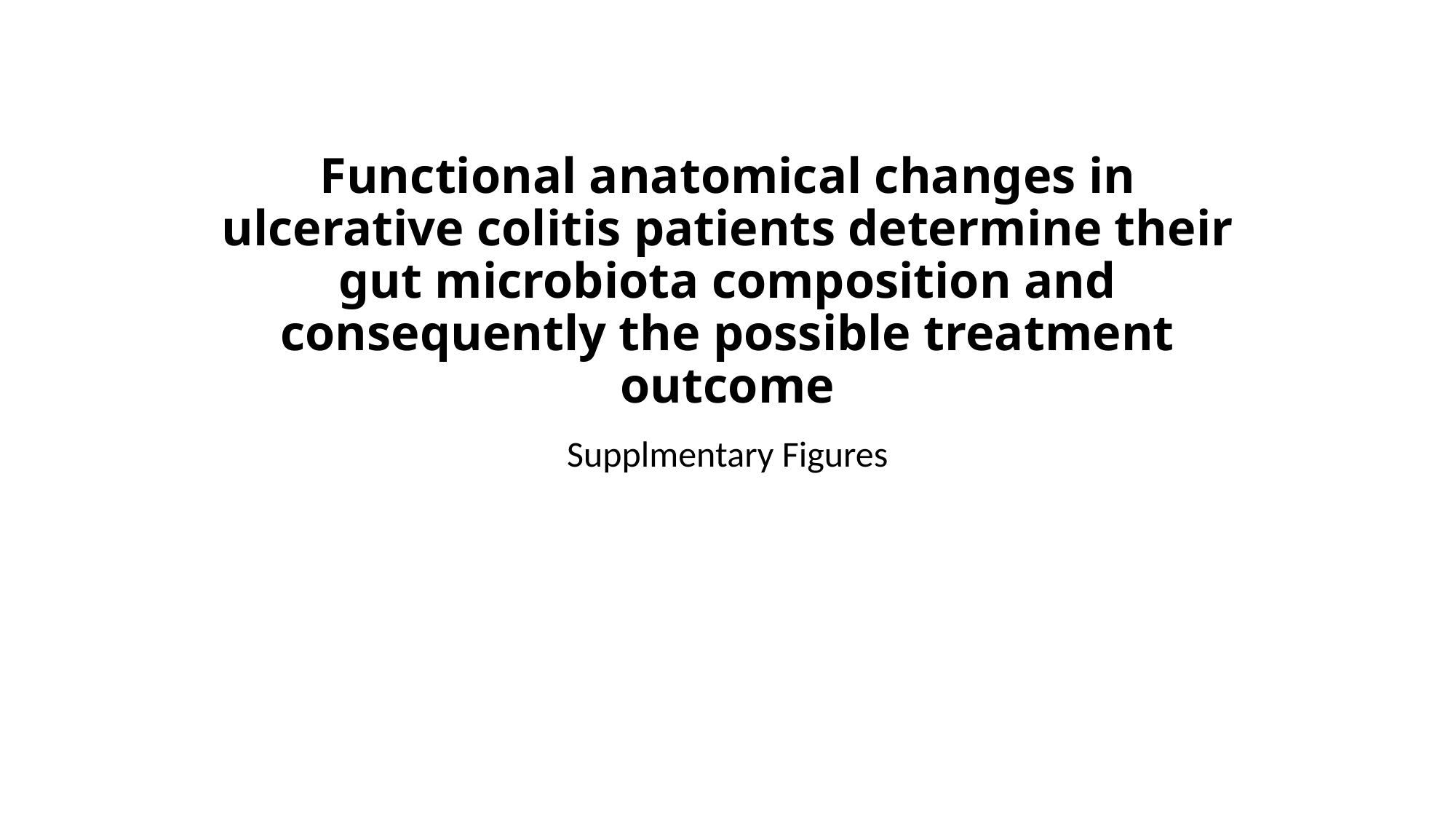

# Functional anatomical changes in ulcerative colitis patients determine their gut microbiota composition and consequently the possible treatment outcome
Supplmentary Figures

## Slide 2
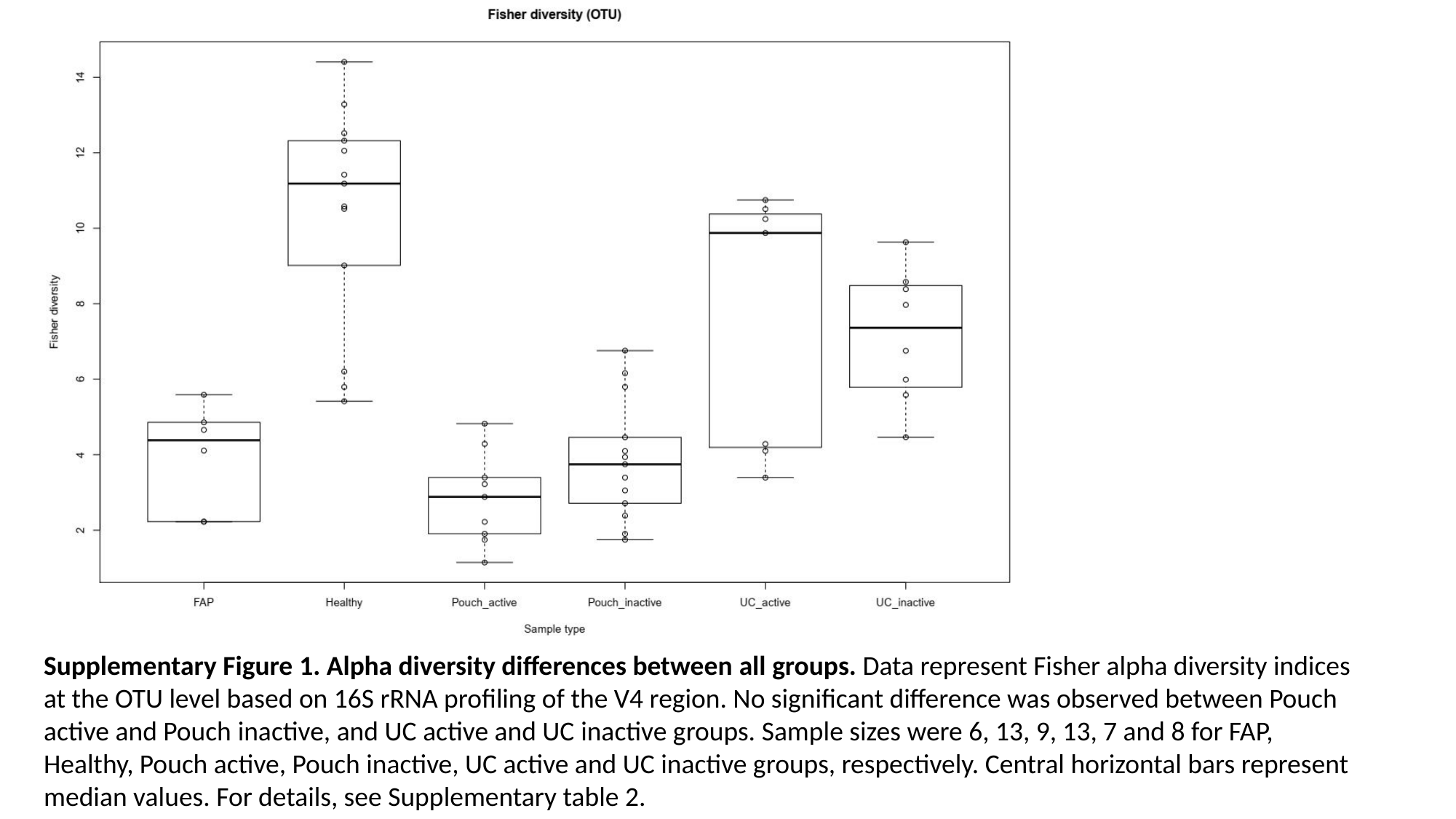

Supplementary Figure 1. Alpha diversity differences between all groups. Data represent Fisher alpha diversity indices at the OTU level based on 16S rRNA profiling of the V4 region. No significant difference was observed between Pouch active and Pouch inactive, and UC active and UC inactive groups. Sample sizes were 6, 13, 9, 13, 7 and 8 for FAP, Healthy, Pouch active, Pouch inactive, UC active and UC inactive groups, respectively. Central horizontal bars represent median values. For details, see Supplementary table 2.

## Slide 3
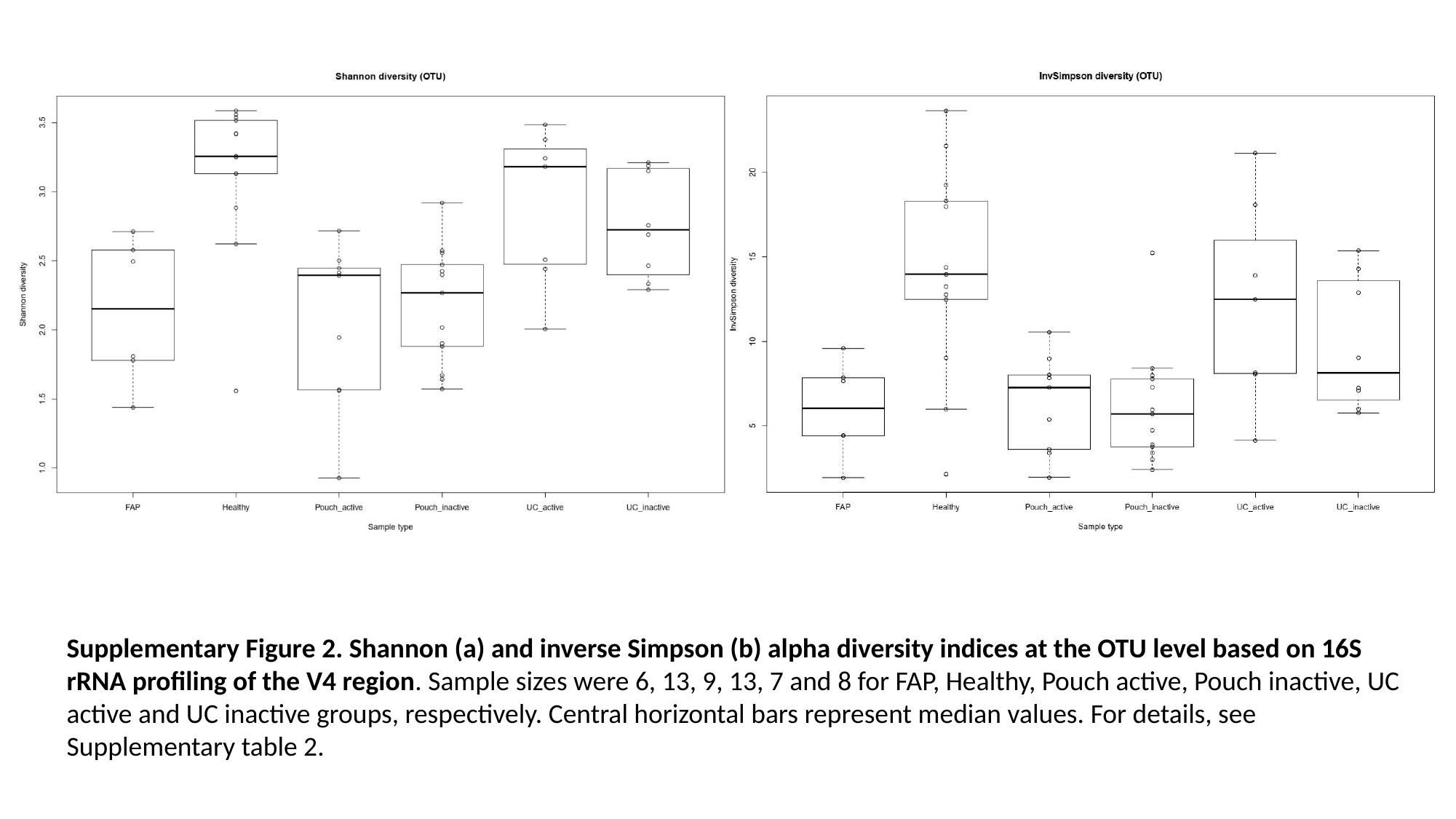

Supplementary Figure 2. Shannon (a) and inverse Simpson (b) alpha diversity indices at the OTU level based on 16S rRNA profiling of the V4 region. Sample sizes were 6, 13, 9, 13, 7 and 8 for FAP, Healthy, Pouch active, Pouch inactive, UC active and UC inactive groups, respectively. Central horizontal bars represent median values. For details, see Supplementary table 2.
